# The impact of continuous and intermittent supportive counseling on self-efficacy and continuation of breastfeeding in lactating women affected by COVID-19: a quasi-experimental trial

**DOI:** 10.1186/s12884-024-06572-2

**Published:** 2024-05-17

**Authors:** Maryam Karimi, Azam Maleki, Leila Rastegari

**Affiliations:** 1 Department of Midwifery, School of Nursing and Midwifery, Zanjan University of Medical Sciences, Zanjan, Iran; 2 Social Determinants of Health Research Center, Health and Metabolic Diseases Research Institute, Zanjan University of Medical Sciences, Zanjan, Iran; 3 Social Determinants of Health Research Center, Health and Metabolic Diseases Research Institute, Zanjan University of Medical Sciences, Azadi Square, Jomhori Eslami St, Zanjan, 4515613191 Iran

**Keywords:** Breastfeeding self-efficacy, Breastfeeding continuity, Counselling, Covid-19

## Abstract

**Background:**

Promoting exclusive breastfeeding can have a great effect in reducing the complications and mortality rate of mother and child.

**Objective:**

The study aimed to compare the effects of continuous and intermittent supportive counselling on the self-efficacy and continuity of breastfeeding among Lactating mothers with COVID-19.

**Methods:**

The study was a semi-experimental research method and was conducted on 73 mothers with COVID-19 who were hospitalized in Ayatollah Mousavi Hospital in Zanjan, Iran from May 2021 to April 2022. In the continuous counselling group, counselling was provided daily for 14 days, while in the intermittent counselling group, counselling was provided once a week for four weeks. Breastfeeding continuity was assessed based on the World Health Organization’s classification, and breastfeeding self-efficacy was measured using Dennis’ standard breastfeeding self-efficacy questionnaire (BSE) up to four months after delivery. The data were analyzed using chi-square tests, independent t-tests, paired t-tests, analysis of variance with repeated measures, and survival analysis (Kaplan-Meier) with a 95% confidence level.

**Results:**

The survival analysis revealed that the cessation of exclusive breastfeeding occurred in 17 cases within the continuous counselling group and in 22 cases within the intermittent counselling group. The rates of continuation for exclusive breastfeeding were 52.8% and 40.5% in the continuous and intermittent counselling group respectively. However, no statistically significant differences were observed in the continuation of breastfeeding and the trend of changes in the mean scores of breastfeeding self-efficacies between the continuous and intermittent counselling groups. Furthermore, comparing the change in breastfeeding self-efficacy scores between the one-month and four-month follow-ups within the continuous counselling group, a statistically significant increase was observed.

**Conclusion:**

The results indicated no difference in the effectiveness of continuous and intermittent counseling methods in improving breastfeeding continuity in women with COVID-19. Further research is needed to explore the long-term effects of different counseling approaches on breastfeeding outcomes during crises.

**Trial registration:**

The study was registered on the Iranian Registry of Clinical Trials website on 29/06/2021 with the registration code IRCT20150731023423N19. It can be accessed via this link: https://irct.behdasht.gov.ir/user/trial/55391/view.

## Introduction

The COVID-19 pandemic has impacted health in various ways; one being the quality and quantity of exclusive breastfeeding. The release of initial findings on the potential risk of COVID-19 transmission through direct contact, and concerns about transmitting the disease to newborns, posed challenges to breastfeeding [1]. In a systematic review, the prevalence of exclusive breastfeeding in mothers with COVID-19 was 56.76%. Based on the year of publication, the analysis indicated that the average breastfeeding rate was 49.78% in studies from 2020, which was lower than the 68.39% in 2021. This implies a decline in breastfeeding rates during the COVID-19 outbreak compared to the post-COVID-19 period [2]. In another study, Nismath et al. discovered that mothers with COVID-19 had notably lower breastfeeding self-efficacy [3]. Inconsistent findings have also been documented, as evidenced in a study by Lapillonne et al. indicating that breastfeeding rates rose during the Covid-19 outbreak compared to pre-coronavirus times [4]. 

Concerns about the virus being transmitted through breast milk have led some mothers with COVID-19 to avoid breastfeeding [5]. However, a study found that formula-fed babies had a higher rate of positive COVID-19 tests compared to breastfed babies [6]. Another reason for the decline in breastfeeding rates was the concern and anxiety brought on by the restrictions imposed due to the spread of the COVID-19 disease in society, which impacted all segments of the population, including pregnant and lactating women [7]. The impact of maternal anxiety on breastfeeding self-efficacy is well-documented in a study [8]. During the initial phase of the pandemic, parents faced challenges in accessing lactation support, struggled to meet breastfeeding goals, and encountered barriers in seeking help. However, in the later stages of the pandemic, parents had fewer interruptions in professional support and increased access to virtual services [9]. The support of employers in critical situations, such as during the COVID-19 pandemic, plays a crucial role in increasing the sense of security, and self-confidence, and reducing the stress of mothers [10, 11]. The COVID-19 pandemic has resulted in a significant rise in remotely delivered maternity care services, such as breastfeeding support. Remote interventions can effectively enhance exclusive breastfeeding in comparison with standard or usual care [12]. 

A meta-analysis study has underscored the positive impact of training or counselling interventions utilizing individual, group, or family-oriented approaches, whether grounded in theoretical frameworks or traditional methods, in enhancing self-efficacy and promoting breastfeeding continuity [13]. The utilization of telephone counselling has been introduced in certain studies due to its availability and convenience. This approach allows for remote support, extending accessibility to a broader spectrum of individuals, including those facing challenges in accessing face-to-face counselling [14, 15]. Moreover, in various studies, implementing protocols for continuous or intermittent breastfeeding counselling via video calls or phone calls has shown promising results in enhancing breastfeeding self-efficacy and continuity for both full-term and preterm infants [16–18]. Despite these encouraging findings, uncertainties persist regarding the optimal delivery methods for counselling sessions. Questions remain about the effectiveness of conducting counselling sessions face-to-face, online, or through phone-based platforms for training purposes, as well as determining the most effective approach for ensuring continuity through continuous or intermittent sessions, particularly in developing countries with low digital literacy and limited Internet connectivity [12, 19]. Further exploration and research are essential to address these uncertainties and establish best practices in the realm of breastfeeding support and education. Due to the spread of the new coronavirus, many breastfeeding support counselling services have transitioned from face-to-face sessions to online forms [20]. There may be a knowledge gap regarding the effectiveness of various executive guidelines, including continuous and intermittent counselling, in improving breastfeeding outcomes especially in low- and middle‐income countries [12, 14, 15] This research aims to compare the effects of continuous and intermittent supportive counselling on the self-efficacy and continuation of breastfeeding in mothers with COVID-19. The study intends to fill the knowledge gap in understanding the effectiveness of different counselling approaches for breastfeeding support in this specific population.

## Method

### Study design and setting

The study was a semi-experimental research method and was conducted on mothers with COVID-19 who were hospitalized in Ayatollah Mousavi Hospital in Zanjan, Iran from May 2021 to April 2022. This study aimed to compare the effects of continuous and intermittent support counselling on the self-efficacy and continuity of breastfeeding in mothers with COVID-19. The study took place in an isolated ward for pregnant mothers with COVID-19 at Ayatollah Mousavi Zanjan Hospital. Ayatollah Mousavi Zanjan Hospital being a tertiary hospital indicates that it is a specialized medical facility that provides advanced medical services, including specialized care for high-risk cases such as pregnant mothers with COVID-19.

### Participants

The research included all mothers who gave birth while hospitalized in the ward. The sample size was determined based on a previous study by Harris Luna et al., considering (p1 = 0.45, p2 = 0.13, 80% power and 95% confidence) 32 participants per group, accounting for a 15% drop-out rate. The final sample size included an additional 37 participants in each group [21]. 

The inclusion criteria of mothers include the desire to participate in the study, having a smartphone with the ability to use WhatsApp, having a definite infection with COVID-19 based on a positive PCR test or CT scan result, the general condition of the mother being favourable to start feeding the baby after delivery, hospitalization in the ward at least 24 h after delivery. The criteria for the inclusion of newborns included a healthy newborn the ability to feed with breastmilk and a gestational age at birth of more than 34 weeks. Exclusion criteria included delivery less than 34 weeks of pregnancy, maternal or infant contraindications for breastfeeding, hospitalization of the infant or mother in the intensive care unit, and unwillingness to continue cooperation.

### Procedure

The eligible participants for the study were selected using an available sampling method. After verifying the inclusion and exclusion criteria, they were divided into two intervention groups, namely continuous counselling and intermittent counselling through a coin toss.

The content of the breastfeeding counselling was adjusted based on the protocol and guidelines of the Ministry of Health, as well as the previous study conducted by the research team [16]. In Iran, breastfeeding counselling was routinely provided in hospitals during the postpartum phase to all mothers, regardless of whether they had COVID-19 infection. However, ongoing counselling after discharge was not included in the standard practice. Following the World Health Organization’s recommendations (March 18, 2020) to initiate breastfeeding within the first hours after birth for women with COVID-19, while observing proper respiratory precautions, this protocol was also adopted in Iran for mothers and babies in good general health. Nevertheless, in practice, some doctors and parents opted out of this practice. In this study, “counselling” refers to personalized interactions between women and midwives, focusing on tailored support and guidance.

In Iran, as in many other countries, standard postpartum care includes breastfeeding education programs immediately after childbirth in a hospital. Postpartum routine care at health centers involves three visits on days 3, 15, and 40 after birth. The key counseling topics cover personal hygiene, breastfeeding, immunization, vitamin use, postpartum hemorrhage or infection examinations, baby care, family planning, and nutrition. However, due to the COVID-19 pandemic, face-to-face visits were limited, following COVID-19 health protocols. Additionally, at the onset of the pandemic, mothers and newborns were separated after childbirth.

The first author, who had completed relevant courses on breastfeeding at Ayatollah Mousavi Hospital in Zanjan, was responsible for implementing the counselling protocol. This ensures that the counselling sessions are conducted by a trained professional with expertise in breastfeeding support. In both groups, the first session of breastfeeding counselling was conducted face-to-face and individually. This session took place in the hospital, following the health protocols for COVID-19, and lasted for 45 min. The counselling session was held at the patient’s bedside. Following the initial session, the continuous supportive counselling group received daily counselling for 14 days. This counselling was conducted through phone calls and the delivery of educational content via WhatsApp. In the intermittent supportive counselling group, counselling sessions occurred once a week for a total of four weeks. Similar to the continuous group, counselling in this group was also delivered through phone calls and the transmission of educational materials via WhatsApp. Additionally, as part of the counselling process, mothers in both groups had the opportunity to ask questions and receive answers by sending messages on WhatsApp.

During the first session of breastfeeding counselling, the following activities were conducted:

(1) Self-introduction and getting to know the patient, (2) Explanation of the objectives of the study, (3) Definition of exclusive breastfeeding and its benefits for the baby, (4) Explanation about the new coronavirus disease and concerns of mothers with COVID-19 regarding breastfeeding, (5) Health recommendations for infected mothers with COVID-19 during breastfeeding, (6) Explanation about the number and frequency of breastfeeding throughout the day, (7) Explanation of how to breastfeed and observing mothers breastfeeding based on the Latching-on Checklist, (8) Answering mothers’ questions regarding breastfeeding or COVID-19. In this study, emotional support was provided to mothers who expressed fears and concerns about COVID-19 transmission through breastfeeding.

The counsellor collected the mothers’ contact information for future counselling sessions and conducted a pre-test.

During the subsequent phone call sessions and the delivery of educational content via WhatsApp, the focus of the counselling and educational materials was on the following topics:

(1) Discussing fears and concerns of breastfeeding mothers in the era of COVID-19, (2) Explaining misconceptions of breastfeeding in the era of COVID-19, (3) Explaining the benefits of breastfeeding for babies, (4) Explaining the assessment of breast milk adequacy, (5) Explaining the risks of formula feeding, cow’s milk and milk alternatives, (6) Health recommendations for mothers with COVID-19 while breastfeeding, (7) Teaching various correct breastfeeding techniques, (8) Strategies to increase breast milk production, including recommendations for adequate nutrition, hydration, and breastfeeding frequency, (9) Preventing and solving breast problems such as engorgement or mastitis, and guiding prevention and management, (10) The importance of breastfeeding during the night and its role in maintaining milk supply, 11. How to use supplements for the baby such as vitamin D, 12. Encouragement to take care of the baby with the support and participation of the family.

### Outcomes

The primary outcome of the study was the continuation of breastfeeding and the second outcome was breastfeeding self-efficacy. Breastfeeding self-efficacy was measured at three-time points: before counselling, four weeks after delivery, and four months after delivery. Additionally, the continuation of breastfeeding was monitored monthly until four months after delivery.

## Data collection tools

### Demographic characteristics

This checklist included the participants’ age, education level, occupation, place of residence, family income, number of previous pregnancies, and whether the current pregnancy was wanted or unwanted. Additionally, it included details regarding the skin-to-skin contact between the mother and baby in the first hour after birth, gestational age at delivery, and the type of delivery method.

### The Breastfeeding Self-Efficacy Scale-Short Form (BSES-SF)

The Dennis breastfeeding self-efficacy questionnaire consisted of 14 items designed as self-report questions. Each question began with the phrase “I always can” and was rated on a 5-point Likert scale. The response options ranged from 1 (indicating “never or not at all sure”) to 5 (indicating “I am completely sure”). The total score of the questionnaire ranged from 14 to 70, with a higher score indicating higher breastfeeding self-efficacy [22]. In a study conducted by Amini et al. in 2018, the psychometrics of the Persian version of the Breastfeeding Self-Efficacy Questionnaire were examined. The reliability of the questionnaire was assessed using Cronbach’s alpha coefficient, which was found to be 0.91, indicating high internal consistency. Additionally, the validity indicators of the questionnaire’s structure were found to be in good condition, suggesting that the questionnaire effectively measured breastfeeding self-efficacy in the Iranian context [23]. In the present study, the reliability of the questionnaire was assessed, and it was confirmed to be highly reliable with a Cronbach’s alpha coefficient of 0.94.

### Continuity breastfeeding

A breastfeeding classification system has been introduced by the World Health Organization [24]. In this study, a classification system was used to interpret the results of breastfeeding continuation. The classification system consisted of three levels: exclusive breastfeeding, combined breastfeeding (50% breast milk and 50% formula), and bottle feeding (100% formula). These classifications were used to better understand and analyze the patterns of breastfeeding practices in the study population.

### Statistical analysis

In this research, the data analysis was conducted using SPSS 16 software. The researchers employed various statistical tests to analyze the data and determine the significance of the findings. Firstly, the Chi-square test was used to compare demographic characteristics, qualitative variables, and breastfeeding patterns between the two groups. Next, the Kolmogorov-Smirnov test was used to assess the normal distribution of the data. To compare breastfeeding self-efficacy before and after the intervention within the groups, the paired sample t-test was used. The independent t-test was used to compare breastfeeding self-efficacy between the two groups. A repeated measure (ANOVA) was used to measure the effect of time and the interaction effect of time and group. Finally, the Kaplan-Meier survival analysis method was used to measure the continuation of breastfeeding. A significance level of 0.05 was considered.

## Results

**Fig. 1:**
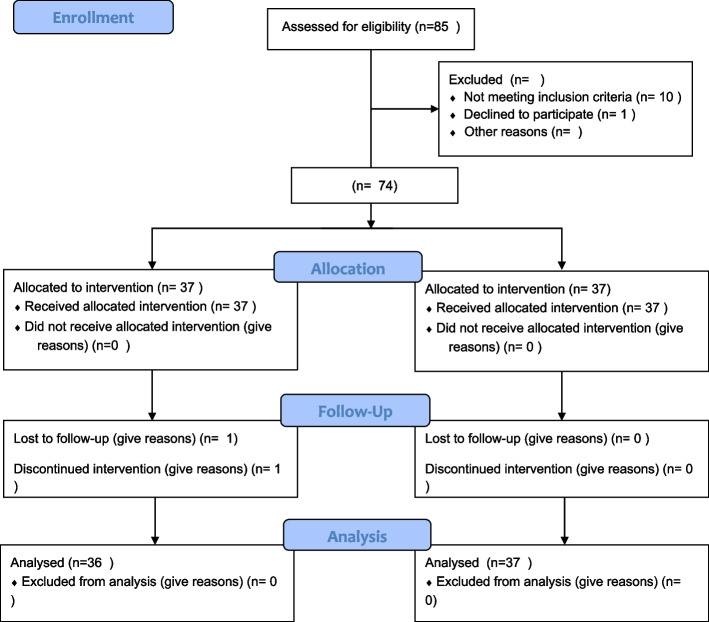
The process of participant enrolment

In this study, a total of 85 individuals were initially examined for eligibility. However, 7 individuals were excluded due to gestational age less than 34 weeks, 3 individuals were excluded because their babies were hospitalized in the neonatal intensive care unit, and 1 individual declined to participate. As a result, a total of 74 individuals (34 in each group) were included in the study (Fig. 1).

In the continuous supportive counselling group, one individual was further excluded due to complications related to COVID-19. Therefore, the findings presented in this section are based on the analysis of data from 73 mothers with COVID-19.

### Baseline data

The results of the chi-square test indicated that there were no significant differences between the two groups in terms of demographic characteristics (Table 1).

**Table 1:** The comparison of demographic characteristics of the participants in terms of two groups

Variables		Continuous Group		Intermittent Group		*P* Value
		Frequency	%	Frequency	%	
Sample Size	73	36	100	37	100	
Age (Year)	17–20	6	16.7	3	8.1	0.142
	21–25	8	22.2	8	21.6	
	26–30	14	38.9	23	62.2	
	31–35	8	22.2	3	8.1	
Education	Primary	9	25	2	5.4	0.118
	Secondary	8	22.2	9	24.3	
	High School	5	13.8	4	10.8	
	Diploma	10	27.8	12	32.4	
	University	4	11.1	10	27	
Job	Employee	1	2.8	5	13.5	0.095
	No Employee	35	97.2	32	86.5	
Place of Residence	Urban	22	81.1	22	59.5	0.885
	Rural	14	38.9	15	40.5	
The Amount of Income	Adequate	4	11.1	11	29.7	0.125
	Less Than Enough	9	25	9	24.3	
	Moderate	23	63.9	17	45.9	
Desired Pregnancy	Yes	32	88.9	29	78.4	0.226
	No	4	11.1	8	21.6	
Gestational Age	34–36	7	19.4	8	21.6	0.818
	37–40	29	80.6	29	78.4	
Type of Delivery	Normal Vaginal Delivery	17	47.2	16	43.2	0.733
	Cesarean Section	19	52.8	21	56.8	
Gravida	1	16	44.4	15	40.5	0.726
	2–5	20	55.6	22	59.5	
Skin-To‐Skin Contact	Yes	14	38.9	12	32.4	0.565
	No	22	61.1	25	67.6	

### Continuity breastfeeding

The percentage of exclusive feeding in the continuous counselling group was 61.1% in the first month, while it was 45.9% in the intermittent counselling group. However, the results of the Chi-square test indicated that there was no statistically significant difference between the two groups in terms of breastfeeding patterns in the first, second, third, and fourth months after delivery (Table 2).

**Table 2:** The comparison of breastfeeding patterns of the participants in terms of two groups

Breastfeeding Patterns		Continuous Group		Intermittent Group		*P* Value
Sample size	73	Frequency	%	Frequency	%	
In the First Month	BMF	22	61.1	17	45.9	0.303
	Mix	14	38.9	19	51.4	
	Bottle	0	0	1	2.7	
Second Month	BMF	28	77.8	23	62.2	0.139
	Mix	8	22.2	11	29.7	
	Bottle	0	0	3	8.1	
The Third Month	BMF	27	75	22	59.5	0.140
	Mix	9	25	12	32.4	
	Bottle	0	0	3	8.1	
The Fourth Month	BMF	25	69.4	25	67.6	0.911
	Mix	9	25	9	24.3	
	Bottle	2	5.6	3	8.1	

*BMF* Breast Milk Feeding, Mix (BMF+ Bottle)

The survival analysis, specifically the Kaplan-Meier estimate, was used to analyze the cessation of exclusive breastfeeding in both the continuous counselling group and the intermittent counselling group (Table 3). The results showed that there were 17 cases of cessation in the continuous counselling group and 22 cases in the intermittent counselling group. The continuation of exclusive breastfeeding was found to be 52.8% in the continuous counselling group and 40.5% in the intermittent counselling group. This indicates that a higher percentage of participants in the continuous counselling group continued exclusive breastfeeding compared to the intermittent counselling group. Furthermore, the average duration of exclusive breastfeeding until the fourth month of follow-up was 86.19 days in the continuous counselling group and 70.48 days in the intermittent counselling group. However, the difference in the average duration of exclusive breastfeeding between the two groups was not statistically significant (Table 3).

**Table 3:** The Survival analysis of the continuation of exclusive breastfeeding after four months of childbirth in two groups

Group	Cessation BMF	BMF		Mean (day)	SE	95% Confidence Interval		*P* value
	Number	Number	%			Low	High	
Continuous Group	17	19	52.8	86.19	7.31	71.86	100.52	0.251
Intermittent Group	22	15	40.5	70.48	8.05	54.70	86.26	
Total	39	34	46.6	78.64	5.56	67.73	89.55	

Standard error, *BMF* Breast Milk Feeding

**Fig. 2:**
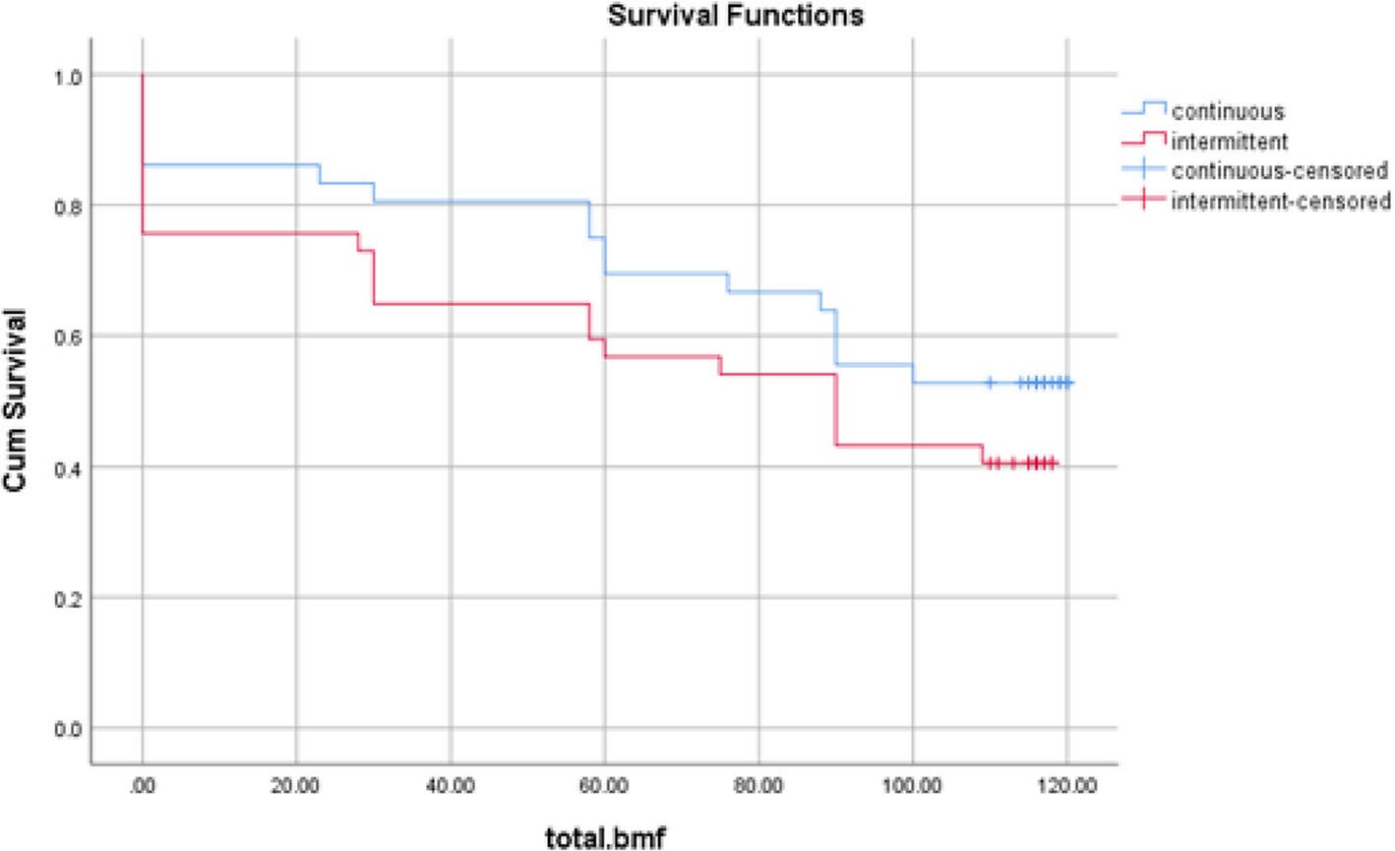
Changes BMF in two groups based on Kaplan Mayer Survival Analysis

The majority of mothers in both groups, specifically more than 78%, initiated exclusive breastfeeding from the first day after delivery. However, there was a decline in exclusive breastfeeding observed in multiple periods, including the first, second, and third months after delivery. In the intermittent counselling group, the highest drop in exclusive breastfeeding occurred on day 90. On the other hand, in the continuous counselling group, the highest drop in exclusive breastfeeding was observed on day 110. This suggests that there was a longer duration of exclusive breastfeeding in the continuous counselling group compared to the intermittent counselling group. Figure 2 likely provides a visual representation of the decline in exclusive breastfeeding over time for both groups (Fig. 2).

### Breastfeeding self-efficacy

The average breastfeeding self-efficacy score in the continuous counselling group showed an increase from 38.27 before counselling to 41.33 four months later. In contrast, the average self-efficacy score in the intermittent counselling group was 38.54 before counselling, which decreased to 38.11 four months after counselling. However, this change was not statistically significant.

The researchers used a repeated measure ANOVA test to examine the changes in average breastfeeding self-efficacy in both the continuous counselling group and the intermittent counselling group. The results showed that the adjusted average of breastfeeding self-efficacy changes in the continuous counselling group was 39.32 ± 1.24, while in the intermittent counselling group it was 37.51 ± 1.32. However, this difference was not statistically significant (*F* = 0.993, *P* = 0.323). Additionally, the interaction between time and group was not significant (*F* = 0.885, *P* = 0.424), indicating that there was no major difference in the changes over time between the two counselling groups. In other words, the trend of changes in breastfeeding self-efficacy did not significantly differ between the continuous counselling group and the intermittent counselling group (Table 4).

**Table 4:** The repeated measure test results mean scores of BSE comparison between two study groups

	Mean ± SD (before)	Mean ± SD (First month)	Mean ± SD (Four months)	Estimated Marginal Means ± SE	Repeated measure test		
					Within subject	Between group	Time * group
**Continuous**	38.27 (11.08)	38.36 (9.38)	41.33 (8.03)	39.32 ± 1.24	*F* = 0.877 *P* = 0.415 Eta = 0.013	*F *= 0.993 *P* = 0.323 Eta = 0.015	*F* = 0.855 *P* = 0.424 Eta = 0.013
**Intermittent**	38.54 (10.20)	36.51 (10.25)	38.11 (10.16)	37.51 ± 1.32			
*P*-value*	0.916	0.438	0.140				

* Independent t-student, *BSE* Breastfeeding Self-Efficacy, *SD* Standard deviation

* Repeated measures ANOVA, Mauchly’s Test of Sphericity (Mauchly’s W = 0.919, *p* = 0.065)

The paired t-test analysis revealed that there were no significant differences in breastfeeding self-efficacy scores in the intermittent counselling group between the one-month and four-month follow-up periods compared to before the intervention. However, in the continuous counselling group, there was a statistically significant decrease in self-efficacy scores from the one-month follow-up to the four-month follow-up (Table 5).

**Table 5:** Intragroup comparison of breastfeeding self-efficacy score before and after counseling (paired t-test)

Group			MD	SE	95% Confidence Interval		T	*P* Value
					Lower	Upper		
Continuous	Pair 1	Pre - Post1	-0.08	1.88	-3.91	3.74	-0.04	0.965
	Pair 2	Pre – Post2	-3.05	1.75	-6.61	0.49	-1.74	0.090
	Pair 3	Post1 - Post2	-2.97	1.44	-5.89	-0.04	-2.06	**0.046**
Intermittent	Pair 1	Pre - Post1	1.45	2.28	-3.20	6.11	0.63	0.529
	Pair 2	Pre – Post2	0.27	2.10	-3.99	4.55	0.13	0.896
	Pair 3	Post1 - Post2	-0.31	1.77	-3.94	3.31	-0.17	0.862

*MD *Mean Difference, *SE *Standard Error

## Discussion

The present study aimed to compare the effectiveness of continuous and intermittent counselling methods in improving exclusive breastfeeding continuation and self-efficacy in hospitalized women with COVID-19. The results showed that the continuation of exclusive breastfeeding was 52.8% in the continuous counselling group and 40.5% in the intermittent counselling group. However, the difference in continuation of exclusive breastfeeding between the two groups was not statistically significant.

In a review of 29 articles, Gavine et al. found that remote breastfeeding support and education, along with hospital support, effectively increased exclusive breastfeeding rates at 3 months [12]. Our findings contrast with those of Gavine’s study. In Gavine’s review, most comparisons in the studies were made against standard or usual care, and the frequency of interventions varied across the studies. This diversity in intervention frequency may have contributed to differing outcomes between our study and Gavine’s review. Also, breastfeeding support is complex and there may be important elements that are not easily addressed remotely. Factors such as the heightened levels of stress and fatigue experienced by individuals as a result of the COVID-19 pandemic, potential separation of mother and child post-childbirth, the mode of delivery, and concerns about infection risk could significantly disrupt the continuity of breastfeeding among women impacted by the virus. These multifaceted challenges could present formidable barriers to the success of remote breastfeeding support interventions, thereby contributing to the differing outcomes observed between our study and Gavine’s review.

It was noted that there were no available studies specifically comparing intermittent and continuous counselling in lactating women with COVID-19. However, the findings of this study were consistent with previous research conducted before the COVID-19 pandemic, suggesting that the results are in line with existing evidence. In Tahir et al.‘s study, the implementation of telephone counselling provided in the first-month post-delivery could increase exclusive breastfeeding rates. This finding suggests that early intervention through telephone support can have a positive impact on promoting exclusive breastfeeding during the initial stages postpartum. However, despite the initial success observed in the first month, the study did not find a significant difference in exclusive breastfeeding rates during the fourth and sixth months after delivery. This could imply that the effects of telephone counseling may diminish over time or that additional or different intervention may be necessary to sustain exclusive breastfeeding practices beyond the immediate postpartum period. Further research and exploration may be needed to determine the most effective strategies for promoting and maintaining exclusive breastfeeding throughout the entire duration of the breastfeeding journey [14]. 

In the present study, only 78% of mothers in both groups started exclusive breastfeeding from the first day after delivery. The study observed a decline in exclusive breastfeeding rates at various time points, including the first, second, and third months after delivery. The largest drop in exclusive breastfeeding was observed on the 90th day in the intermittent counselling group and on the 110th day in the continuous counselling group. The timing of starting breastfeeding immediately after childbirth in women with COVID-19 can depend on the general condition of the affected women or the implementation of the instruction to separate mother and child to prevent the transmission of the disease from mother to baby. Additionally, factors such as increased elective cesarean deliveries, hospitalization of the baby or mother, and breastfeeding problems during the postpartum period affected the mother’s ability to breastfeed [25]. According to Latorre et al.‘s study, the implementation of health and quarantine protocols had a detrimental effect on the continuation of exclusive breastfeeding among non-COVID-19 mothers [26]. A similar finding was also reported in the study conducted by Oggero et al., further highlighting the negative impact of health and quarantine protocols on exclusive breastfeeding continuation among non-COVID-19 mothers. The results from both studies suggest that the disruptions caused by the pandemic-related measures have posed significant challenges for mothers who are striving to exclusively breastfeed their infants [27]. 

According to the findings of the present study, there was no statistically significant difference in breastfeeding self-efficacy scores between the two groups. Additionally, there was no significant difference in the trend of changes in breastfeeding self-efficacy between the two groups. However, within the continuous counselling group, there was a significant increase in breastfeeding self-efficacy scores at the one-month follow-up compared to the four-month follow-up. Dağlı et al.‘s study found that implementing continuous remote breastfeeding education during the COVID-19 epidemic was effective in improving breastfeeding self-efficacy among mothers for up to six months after delivery [28]. Indeed, the results of the present study are consistent with the findings of the aforementioned studies, indicating that continuous breastfeeding education during the COVID-19 epidemic may be effective in improving breastfeeding outcomes.

In contrast to the findings of the present study, Dodou et al. reported that intermittent telephone counselling, conducted seven, thirty, ninety, and fifty days after delivery, increased breastfeeding self-efficacy in the intervention group [29]. This contradictory result suggests that the effectiveness of intermittent breastfeeding education during the COVID-19 epidemic may vary depending on the specific interventions and timing of counselling sessions. It highlights the importance of considering different approaches and tailoring interventions to individual circumstances and preferences when aiming to improve breastfeeding outcomes during challenging times. The difference in results between the above study and the present study could be attributed to the different intervention methods and the specific challenges posed by the COVID-19 epidemic. The implementation of health protocols during the pandemic has introduced new challenges in terms of changing maternal duties, breastfeeding practices, negative experiences related to breastfeeding, and reduced professional support [30, 31]. 

During the Covid-19 epidemic, a high percentage of pregnant women experienced anxiety symptoms [7, 32]. Physiological responses, such as stress and fatigue, can have an impact on an individual’s self-efficacy. Specifically, individuals who experience high levels of stress tend to have lower levels of self-efficacy [33]. In a study conducted by Nismath et al., it was found that mothers infected with the novel coronavirus had significantly lower breastfeeding self-efficacy scores. Additionally, the fear of virus transmission was identified as a known inhibitory factor in breastfeeding initiation. This fear may have contributed to lower breastfeeding self-efficacy and potentially affected the decision to initiate breastfeeding [3]. Miranda et al. conducted a study that demonstrated the outbreak of the COVID-19 epidemic crisis can lead to depression and insomnia in lactating mothers. These factors, in turn, have a double impact on reducing breastfeeding self-efficacy [34]. 

Based on the findings of the studies mentioned, it appears crucial to implement supportive interventions that target reducing stress and anxiety in lactating mothers during crises like the COVID-19 pandemic. These interventions should be carried out alongside breastfeeding counselling to improve breastfeeding outcomes. By addressing the mental health needs of mothers and providing them with the necessary support, it is possible to enhance breastfeeding self-efficacy and potentially improve overall maternal and child health outcomes. Planners and officials in the field of maternal and child health should consider these findings when designing programs and policies to effectively support mothers during times of crisis.

### Limitation

The study has some limitations that should be taken into account. Firstly, the implementation of the instruction to separate mother and baby after delivery was beyond the control of the researchers. This external factor could have influenced the breastfeeding outcomes and self-efficacy of the participants. Additionally, the study did not measure the level of anxiety experienced by the mothers, which could be an important variable to consider in understanding the impact on breastfeeding self-efficacy. Moreover, due to the critical conditions and limited access to samples during the COVID-19 pandemic, the study was designed as a semi-experimental study. This might have affected the generalizability of the findings to a larger population. Furthermore, it is worth noting that the research was conducted within a specific community of lactating mothers who were infected with Covid-19. This limits the generalizability of the findings to other populations or situations. To gain a more comprehensive understanding of the effectiveness of supportive counselling in improving breastfeeding continuity during crises like the COVID-19 pandemic, it is recommended to conduct further studies that address these limitations. This would provide a clearer view of the impact of counselling interventions on breastfeeding outcomes.

## Conclusion

The results indicated no difference in the effectiveness of continuous and intermittent counseling methods in improving breastfeeding continuity in women with COVID-19. Nonetheless, this study suggests that continuous supportive counseling had a slightly positive impact on enhancing breastfeeding self-efficacy compared to intermittent supportive counseling. Further research is needed to explore the long-term effects of different counseling approaches on breastfeeding outcomes during crises.

## Data Availability

The dataset used in the present study is available from the corresponding author upon reasonable request.
